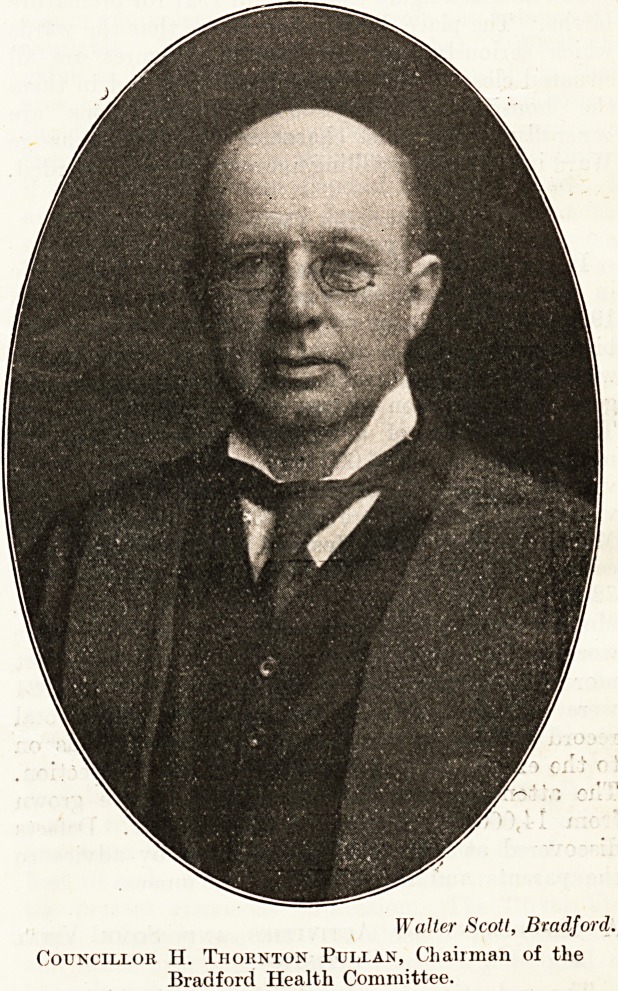# The Public Health: Interviews with Local Authorities

**Published:** 1923-06

**Authors:** 


### THE PUBLIC HEALTH.

### INTERVIEWS WITH LOCAL AUTHORITIES.

### IX.—BRADFORD—THE MUNICIPAL HOSPITAL PIONEERS.

(By our Special Commissioner.)

THEY have a kindly way in Bradford when directing you in reply to your inquiries. The policeman, b fore indicating the way to the Town Hall, takes your arm with an air of gentle familiarity, and the liftman at the Town Hall gives the same sort of friendly clutch before pointing out the corridor which leads to the quarters of the Medical Officer of Health. So we were led to anticipate a kindly reception of our inquiries at the hands of the Chairman of the Public Health Committee, Mr. Pullan, and the Medical Officer, Dr. Buchan. And we were in no sense disappointed.

Dr. John J. Buchan, Medical Officer of Health, Bradford.

*Walter Scott, Bradford.*

Councillor H. Thornton Pullan, Chairman of the Bradford Health Committee.

#### Worstedopolis.

The ubiquitous Leland, in the time of Henry VIII., speaks of "Bradeforde" as "a praty, quik market toune. . . . It standath much by clothing." To-day there are, in the city of Bradford, 351 large factory chimneys, with an average height of 100 ft., and 690 steam boilers in use in the various industries. And, without inquiry, one knows that these are an indication that Bradford still stands much by clothing. Bradford has her engineering works and ironworks, and her mis el-laneous industries, but her main industry, of course, comprises various processes in woollen manufacture—spinning, dyeing, weaving, and, in an especial degree, woolcombing. The whole of the wool used in this country in the manufacture of clothing, carpets, &c., comes into Bradford to be combed. The woollen industry, with its workpeople to a large extent living under sorry housing conditions, and employing a large proportion of women, presents for the constant vigilance of the Health Committee and of their Medical Officer of Health a problem of considerable magnitude and also, in these days of a reluctant public purse, one of great anxiety.

#### Infant Mortality.

Although, in common with the rest of the country, Bradford shows a steady decline in its infant mortality rate, the figures in the last five groups of five years each, from 1896 to 1920, show increases, as compared with the rest of England and Wales, varying from 10 to 25 per cent., though a much better position would be revealed by restricting the comparison to the figures for other large towns. The figures for the five periods for Bradford are 165, 153, 132, 122 and 117. Mr. Pullan and Dr. Buchan are not at all the sort of people to regard this steady decline (or the lower figures for 1921 and 1922) with any complacency, so long as there is any preventable cause of infant mortality to be removed. One or two arresting facts in connection with this sad toll of infant life were brought to our notice. The infantile mortality rate has always been very much higher among illegitimate than among legitimate infants; in some years more than double. The cause distribution of deaths per 1,000 births shows no less a figure than 24·8 in 1921 for premature births. The place distribution shows that the wards which seriously load the mortality figures are all situated close to the centre of the city, and in them the housing and environmental conditions are generally of the worst character. In the Exchange Ward in 1921 the appalling figure of 216 was recorded.

### Protecting Child-Life.

In the fight for the better preservation of child-life in Bradford, valuable work can be recorded. In 1921 nearly 4,000, or 66 per cent., of the registered births, were attended by midwives, who were, almost without exception, trained and working under the general supervision of a woman medical officer. The importance of ante-natal work receives full recognition, nearly 3,000 expectant mothers being visited by either municipal midwives or health visitors in 1921. There were 333 admissions to the Municipal Maternity Hospital and 401 maternity cases admitted to the Municipal General Hospital; 72 cases dealt with by voluntary agency at St. Monica's must not be omitted from the record. The work at the Infant Clinics progresses in such wise that more than 50 per cent. of the infants born in 1921 were brought to the clinics, and there was a total record of 35,784 attendances. This work leads on to the excellent results of school medical inspection. The attendances at the school clinics have grown from 14,000 in 1909 to 75,000 in 1921. Defects discovered at school are followed up by advice to the parents and home visiting by nurses.

### A Miscellany of Activities and Some Vital Statistics.

The reduction in the death-rate of children is reflected in the fall in the general death-rate for the city, to which, of course, many other factors have contributed. Mr. Pullan and his Health Committee, working through and with the advice of a medical officer whose zeal for his work is almost passionate, would indeed feel desperate if, from their activities in connection with the drainage, the sewage disposal, the conversion of privies, the inspection of workshops and bakehouses, the work of inspection under the Shop Acts, the control of smoke, the destruction of rats and mice, the work of controlling the supplies of milk and other articles of food, and from their constant watchfulness in connection with infectious diseases, and especially tuberculosis and venereal disease—if, from all these activities, and from the maternity and child welfare work at which we have more particularly glanced, they did not see definite and encouraging results. These are to be found in a death-rate which had fallen from 25·9 per 1,000 in the bad old days of 1871-75, from 20·9 in 1886-90, and even from 16 in the quinquennium 1916-20, to the figure of 13·9 in 1921.

### The Municipal General Hospital.

The distinctive feature of health administration in Bradford is its Municipal General Hospital. It owes its inception, three years or so ago, to no hostility to the voluntary system. So far from this being the case, the Municipality desires that voluntary effort shall be encouraged—and Mr. Pullan himself has been among the protagonists of voluntary workers—and it is of interest to record that the coming of the Municipal Hospital has, in fact, up to the present, stimulated voluntary effort. But at the time when the Poor Law Infirmary had ceased to function as a military (war time) institution, the Health Committee saw an opportunity, not likely to recur, of remedying the grave scarcity of general hospital beds available for the citizens of Bradford, and with a live public opinion behind them, and not lacking in courage in facing the commitments which such a step would involve, the Council decided to acquire the Poor Law Infirmary and become hospital providers. Voluntary effort in the past had failed to provide, in a general hospital, more than about 200 beds for a city with a population approaching 300,000; the most pressing appeals for public subscriptions had not produced funds adequate to an effective supply of the deficiency in beds. There is to-day a finely-equipped Municipal General Hospital with medical, surgical, children's and maternity wards, in all 600 beds, where those who can pay do pay in accordance with their ability, while the destitute are equally treated; there is no classification, except on medical grounds.

### Back-to-Back Hospital Feeders.

There is unhappily no need to write in detail about the housing problem in Bradford. The fact that there are 40,000 back-to-back houses means that one-half of the whole population are so housed, and it speaks for itself. There will be plenty of cases to feed the General Hospital, the tuberculosis sanatoria, and the graves of the one-year-olds while this condition of things continues in a city which could not properly object to the description of well-to-do. It is a problem calling for the maximum effort of a progressive Council. It has by no means been neglected. Before the war, Mr. Pullan, for the Council, started buying up houses (save the mark!) at £5 and £10 apiece, with a view of course to wiping them out. More recently schemes for the abolition of the worst of the slum areas have been submitted to the Ministry of Health, and in the end those who make up their minds to sting public opinion into activity over this slum question will win through.

## Figures and Tables

**Figure f1:**
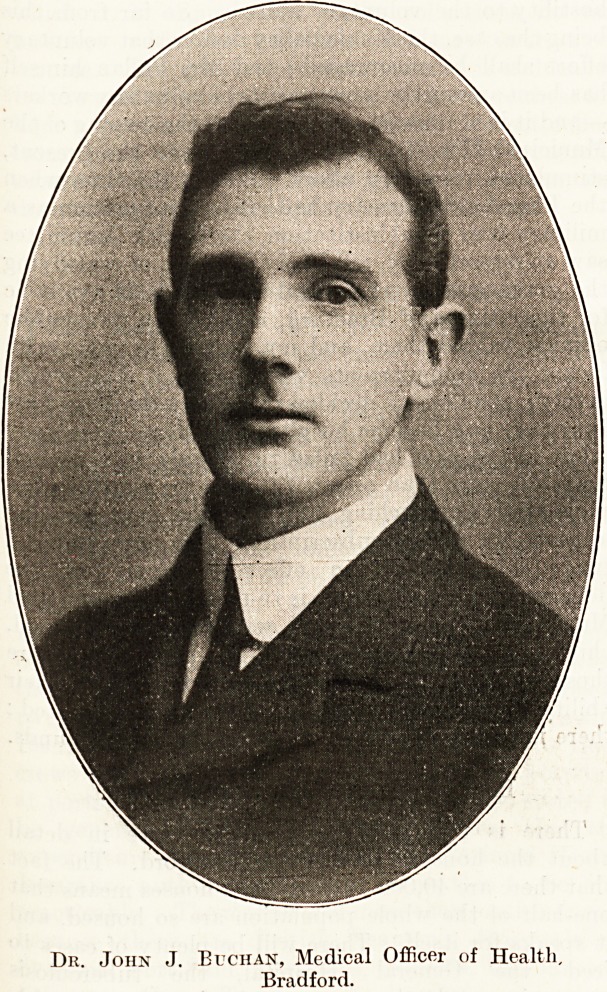


**Figure f2:**